# Modeling development of inhibition zones in an agar diffusion bioassay

**DOI:** 10.1002/fsn3.232

**Published:** 2015-04-27

**Authors:** Vaishnavi Chandrasekar, Stephen J Knabel, Ramaswamy C Anantheswaran

**Affiliations:** Department of Food Science, The Pennsylvania State University202 Rodney A. Erickson Food Science Building, University Park, Pennsylvania, 16802

**Keywords:** Agar bioassay, diffusion, finite element method, nisin

## Abstract

A two-temperature agar diffusion bioassay is commonly used to quantify the concentration of nisin using *Micrococcus luteus* as the indicator microorganism. A finite element computational model based on Fick's second law of diffusion was used to predict the radius of the inhibition zone in this diffusion bioassay. The model developed was used to calculate nisin concentration profiles as a function of time and position within the agar. The minimum inhibitory concentration (MIC) of nisin against *M. luteus* was determined experimentally. The critical time (*T*_c_) for growth of *M. luteus* within the agar diffusion bioassay was experimentally determined using incubation studies with nisin. The radius of the inhibition zone was predicted from the computational model as the location where the predicted nisin concentration at *T*_c_ was equal to MIC. The MIC was experimentally determined to be 0.156 μg mL^−1^, and *T*_c_ was determined to be 7 h. Good agreement (*R*^2^ = 0.984) was obtained between model-predicted and experimentally determined inhibition zone radii.

## Introduction

The use of “all natural” ingredients has received considerable attention from the food industry over the past decade due to consumer preference for natural over chemical preservatives (Smid and Gorris 1999). Nisin is an example of a natural antimicrobial agent that is effective against gram-positive bacteria. This polypeptide is produced during fermentation by strains of *Lactococcus lactis* subsp *lactis* and was granted ‘generally regarded as safe’ (GRAS) status for use in foods by the Food and Drug Administration (FDA) (Hurst 1981). Nisin has significant potential for use as an antimicrobial agent in packaging films and edible films in order to increase the shelf-life of various food products.

Nisin has been quantified using techniques such as reversed phase HPLC (Liu and Hansen 1990) and the bicinchoninic acid protein assay (Were et al. 2004). However, the most widely used method for the quantification of nisin is the one-temperature agar diffusion bioassay (Pongtharangkul and Demirci 2004). In this assay, wells are bored in agar plates seeded with an indicator microorganism. Nisin solution is then added to the wells and nisin is allowed to diffuse. The radius of the zone of inhibition is measured, and this can be correlated back to nisin concentration using a standard curve. The higher the nisin concentration, the larger will be the inhibition zone radius. Several studies have aimed at improving the sensitivity of this assay for better quantification (Rogers and Montville 1991; Wolf and Gibbons 1996; Pongtharangkul and Demirci 2004). A modified agar diffusion method for quantification of nisin was described by Lalpuria et al. (2012). They found that using 3.5 mm diameter wells and prediffusing the nisin solution at 4°C for 48 h followed by incubation at 30°C offered the best precision and sensitivity for nisin quantification, when *Micrococcus luteus* was used as the indicator organism. It would be helpful to develop a model to describe the nisin diffusion phenomena in the two-temperature agar bioassay, to allow for a more complete understanding of the underlying physics in this technique. The model development can also enable us to rapidly construct standard curves required for antimicrobial quantification.

Sebti et al. (2004) studied the diffusion phenomenon of nisin in agarose gels and proposed a mathematical model using Fick's law, to describe it. Using this model, the diffusion coefficient of nisin in agarose gels incubated at varying temperatures was determined, and the diffusion phenomenon was found to satisfy the Arrhenius relationship. Carnet Ripoche et al. (2006) used this model to determine the impact of agarose and fat content on the diffusion coefficient of nisin.

Early attempts have been made to theoretically describe the inhibition zone radii using Fick's laws of diffusion (Cooper and Woodman 1946; Cooper et al. 1958; Awerbuch et al. 1989). The concentration profile of nisin was related to inhibition zone radii using concepts of minimum inhibitory concentration of the antimicrobial (MIC) and critical time of bacterial growth (*T*_c_) as described by Linton (1958). The antibiotic in the well diffuses outward radially during the incubation time. At locations on the agar plate where the antibiotic concentration equals the MIC against the target microorganism, there will be no microbial growth. At locations where the antibiotic concentration is below the MIC, microbial growth will be present. The MIC is also a function of bacterial population in the agar, which continually increases with incubation time. Cooper and Woodman (1946) showed that zones of inhibition were formed at a “definite time” (referred in this paper as critical time) after commencement of incubation of the seeded agar, when the microbial population in the agar becomes higher than what the MIC of nisin can completely inhibit.

Analytical solutions for the diffusion equation with cylindrical coordinates exist when initial conditions are uniform throughout the entire domain space (Crank 1975). This analytical solution can be used to describe the nisin concentration as a function of time in a conventional one-temperature agar diffusion assay. In the more sensitive two-temperature agar diffusion assay, seeded agar plates are initially incubated at 4°C. This step is referred within literature as the ‘prediffusion’ where nisin is allowed to diffuse through the agar before microorganisms start growing. This prediffusion step results in better precision and accuracy of the assay (Lalpuria et al. 2013). The concentration profile of nisin in the agar plate during this prediffusion step can be predicted using an analytical solution. However, during subsequent incubation at the second temperature of 30°C for up to 48 h, the initial conditions are a function of spatial coordinates, and standard analytical solutions cannot be used to predict the concentration of nisin. Numerical modeling using the finite element method (FEM) can instead be employed to predict the diffusion of nisin during the second incubation temperature period.

The FEM is a numerical approach to solve differential equations. In this method, a continuum is divided into many small elements of suitable geometry, and a finite set of points called nodes is associated with each element (Dhatt et al. 2012). This method then employs piecewise continuous functions over the solution region. Several researchers have used the finite element method (FEM) for modeling problems related to heat and mass transfer within food processing (Baerdemaeker et al. 1977; Misra and Young 1979; Zhou et al. 1995; Wang and Sun 2002).

The goal of this study was to develop a FEM to predict the inhibition zone radius in the two-temperature agar diffusion technique described by Lalpuria et al. (2012). The specific objectives were (1) to develop a computational FEM-based model to predict nisin diffusion in agar over time for the two-temperature agar diffusion assay; (2) to experimentally determine the minimum inhibitory concentration (MIC) of nisin and the critical time (*T*_c_) for growth of *M. luteus*; and (3) to theoretically predict the inhibition zone radius using the nisin concentration profile from the model, MIC and *T*_c_ values.

## Materials and Methods

### Materials

*Micrococcus luteus* (ATCC 10240) was grown in Difco nutrient broth (Becton Dickinson and Co., Sparks, MD) at 30°C in an orbital shaker at 150 rpm for 24 h and refrigerated at 4°C before use. The agar media for this assay consisted of 0.8% nutrient broth, 0.75% Bacto agar (Becton Dickinson and Co.), and 1% Tween 20 (BDH®, Solon, OH).

### Mathematical modeling

COMSOL Multiphysics® (Version 4.2, Burlington, MA) was used to develop a three-dimensional model for nisin concentration in the agar plate over time. This software uses the finite element method to solve the differential equations that define various physical processes.

#### Model geometry

**Figure 1:**
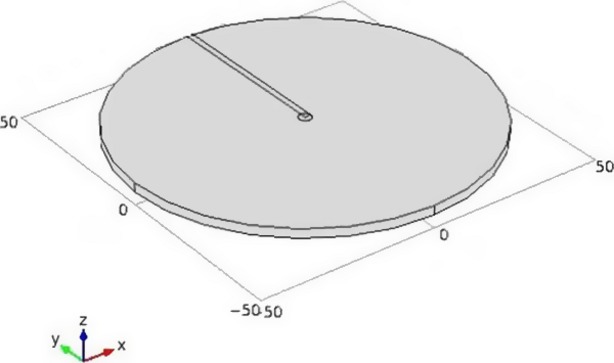
Schematic representation of agar diffusion bioassay, drawn using COMSOL Multiphysics® (Version 4.2, Burlington, Massachusetts).

A simple three-dimensional cylindrical geometry was constructed to represent the agar gel based on experiments conducted as described by Lalpuria et al. (2012), and is shown in Fig.1. Dimensions were based on VWR microbiological petri dishes (100 mm diameter with agar height 2.6 mm). A smaller cylinder was constructed with a diameter of 3.5 mm and height 2.6 mm representing dimensions of the well bored into the agar gel. Additionally, a block was constructed to monitor the concentration of nisin as a function of distance at different times. The model was constructed for the following initial concentrations of nisin in the well: 0.625, 1.25, 2.50, 6.25, 12.50, 18.75, 25, 50, 75, 125 μg mL^−1^.

#### Governing equation and boundary conditions

Sebti et al. (2004) showed that the diffusion of nisin though the agar gel is a mass transfer process that can be described using Ficks second law of diffusion (eq. 1) (Crank 1975).

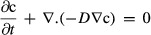
1

where c is the concentration of nisin (mol m^−3^), *D* is the diffusion coefficient or diffusivity of nisin through the agar (m^2^ sec^−1^), *t* is time (sec). Equation 2 was used to solve the steady state nisin flux through the gel.

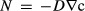
2

where *N* is the steady state permeate flux in mol m^−2^ sec^−1^.

The basic assumption of no flux was used as the boundary conditions for the model geometry in order to simulate the diffusion process. This assumption presumes that no loss of nisin occurs at the gel boundary to the surroundings. The no flux condition was applied to all the boundaries, and can be expressed mathematically as shown in eq. 3:

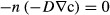
3where *n* is the normal vector to the boundary.

The initial concentration of nisin inside the well was varied between (0.625–125 μg mL^−1^), while concentration in all other domains was set to zero.

The diffusivity of nisin (D) in agarose gels at various temperatures has been calculated by Sebti et al. (2004), and the diffusion phenomenon was found to satisfy Arrhenius relationship. The diffusivity values at 4 and 30°C were calculated by using a regression equation based on their data. The natural logarithm of diffusivity values (*ln D*) was plotted as a function of inverse absolute temperature (1/T), and the linear regression equation was obtained as,

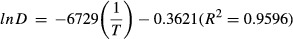
4

Based on eq. 4, the diffusivity of nisin at 4 and 30°C respectively were calculated to be 1.92 × 10^−11^ m^2^ sec^−1^ and 15.77 × 10^−11^ m^2^ sec^−1^, respectively. In our computational model, D_4°C_ was applied at times before 48 h (representing the initial diffusion step) and D_30°C_ was applied at times greater than 48 h up to 96 h (representing the incubation step) with a time step of 1 h.

#### Mesh generation and simulation

**Figure 2:**
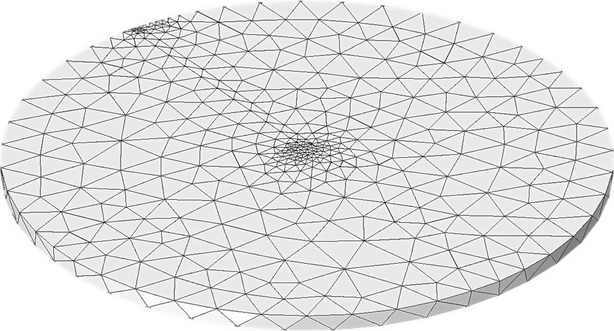
Discretization of the domain into finite elements.

The computational domain was discretized into elements using the meshing operation in COMSOL® (Version 4.2). The size parameter for the elements is an important factor in model simulation. A finer mesh size is capable of producing more accurate results in general, but can take up enormous amount of computation space and time. Hence, an optimum mesh size was determined by repeatedly reducing the element size and analyzing the results from the simulation runs until two subsequent results from the numerical simulation were similar, resulting in convergence. Convergence demonstrates that results of the simulation converge to a finite value and, therefore, are not significantly dependent on the mesh size. A mesh with triangular and tetrahedral elements was created for the domain as shown in Fig.2. A preliminary model was generated using 125 μg mL^−1^ as the initial nisin concentration in the well, and simulations were conducted by consecutively decreasing the element sizes, until the results of two subsequent simulations showed no variation in nisin concentration at the end of the diffusion period, showing convergence. This minimum element size that produced convergence was used in subsequent simulations.

The time-dependent problem was solved by a time-stepping method for each initial concentration of nisin (0.625–125 μg mL^−1^). A user-defined time-dependent solver was used. The ‘variable time step’ (Δ*t*) option was used in COMSOL®, in which small time steps (ranging from 20.8–667.9 sec) were used when the numerical solution is changing very rapidly. Larger time steps (ranging from 1335.8–17,280 sec) were used when the numerical solution is not changing that rapidly. The average simulation time for various initial nisin concentration runs was 2.4 ± 0.3 min.

### Determination of Minimum Inhibitory Concentration (MIC) of nisin against *Micrococcus luteus*

The minimum inhibitory concentration (MIC) of nisin against *M. luteus* was experimentally determined using the two-temperature agar diffusion assay described by Lalpuria et al. (2012). The media for agar bioassay consisted of 0.8% nutrient broth, 0.75% Bacto™ agar, and 1% Tween 20. The agar medium was prepared, autoclaved at 121°C for 30 min, and cooled to 40°C in a water bath. The liquid agar was then inoculated with 1% v/v of 24 h culture of *M. luteus* (approximately 10^7^ CFU mL^−1^). The culture concentration was maintained constant by ensuring that the optical density measured at 600 nm was between 1.6 and 1.7. Fifteen mL of the seeded agar medium was dispensed into petri plates and allowed to solidify at 4°C for 30 min. Holes of diameter 3.5 mm were then bored into the seeded agar plates, and filled with 15 μL nisin solutions of concentrations ranging from 0.625 to 0.009 μg mL^−1^ (prepared by serial dilution). The plates were incubated for 48 h at 4°C followed by incubation for 48 h at 30°C. The plates were examined for the presence of inhibition zones. The inhibition zone (if any) was measured from the edge of the zone with vernier calipers (VWR International Inc., Radnor, PA), and expressed in radial dimensions. Four inhibition zone measurements were taken for each well and averaged. The MIC was determined as the minimum nisin concentration that produced a visually detectable zone of inhibition.

### Determination of Critical Time (*T*_c_) for growth of *Micrococcus luteus*

The critical time (*T*_c_) for growth of *M. luteus* was determined based on the method proposed by Linton (1958). *Micrococcus luteus* was cultured on nutrient broth until the optical density measured at 600 nm was between 1.6 and 1.7 (approximately 10^7^ CFU mL^−1^). One percent seeded nutrient agar plates were prepared as in the MIC experiment. Two holes with diameter 3.5 mm were bored in each plate. Plates were initially incubated at 30°C for different incubation times (1, 2, 3, 4, 5, 6, and 7 h). After the initial incubation, plates were removed, and 15 μL of nisin solution (at a concentration equal to the MIC of nisin, as determined in section 2.3) was added to each well. Plates were again incubated at 30°C for 48 h. Following incubation, the inhibition zone (*z*) was measured using vernier calipers (VWR International Inc., Radnor, PA) and expressed in radial dimensions. Four inhibition zone radii measurements were taken for each well and averaged. The square of the inhibition zone radius was plotted against initial incubation time. The *T*_c_ was determined as the incubation time corresponding to an inhibition zone radius (*z*) value of zero, indicating no inhibition zone.

As a confirmatory test, an experiment was conducted to monitor color of seeded agar plates as an indicator of bacterial growth. *Micrococcus luteus* gives rise to yellow pigmented colonies when grown in agar plates at 30°C (Wieser et al. 2002). Thus in an *L*, *a*, *b* measurement of the seeded agar plates, where ‘*L*’ is the lightness to darkness scale, ‘*a*’ is the red to green scale, and ‘*b*’ is the yellow to blue scale, the degree of yellowness (‘*b*’ values) would be expected to be higher at the end of the experimentally determined critical time as compared to time zero. Nutrient agar medium inoculated with approximately 10^7 ^CFU mL^−1^ *M. luteus* was prepared as described in the section titled “Determination of minimum inhibitory concentration (MIC) of nisin against *M. luteus*”. The agar plates were incubated at 4°C for 48 h followed by incubation at 30°C. Color measurements of the agar surface were made every hour, using a hand-held CR-400 Minolta Chromameter (Minolta Sensing Inc., Tokyo, Japan), and the ‘*b* values’ were measured, which is an indicator of degree of yellowness in the sample. Three measurements were taken from each plate, and the experiment was replicated with five plates at each time point. A two sample *t*-test was used to analyze and compare the data (*α* < 0.05).

Additionally, a growth curve for *M. luteus* in liquid broth was constructed to identify the growth stage at which the critical time coincided, and determine the doubling time of the indicator microorganism. *Micrococcus luteus* cells were inoculated into 200 mL sterile Difco nutrient broth (Becton Dickinson and Co., Sparks, MD) at 30°C in an orbital shaker at 150 rpm for 24 h. Samples were collected every hour and optical density measurements at 600 nm were made with an Eppendorf Bio Photometer (Eppendorf AG, Hamburg, Germany) after appropriate dilutions. The experiment was conducted in triplicate.

### Statistical analysis and model validation

Experiments were replicated, and data were expressed in means ± SD.

The result of the simulation generated concentration profiles of nisin across the agar plate over the entire time period (48 h initial diffusion at 4°C and 48 h incubation at 30°C). The concentration profile at the critical time (*T*_c_) was taken, and the distance over which the concentration of nisin was equal to the MIC was recorded as the predicted zone of inhibition.

These predicted values were compared to the experimentally measured inhibition zones reported by Lalpuria et al. (2013).

## Results and Discussion

### Determination of minimum inhibitory concentration (MIC) of nisin against *Micrococcus luteus*

The average inhibition zone radii for the various concentrations of nisin are shown in Table1. At nisin concentrations 0.078 μg mL^−1^ and below, no inhibition zones were obtained. The MIC of nisin against *M. luteus* is the lowest nisin concentration that produces inhibition, and was determined to be 0.156 μg mL^−1^. This corresponds to a nisin concentration of 4.651 × 10^7^ mol m^−3^ for use in the COMSOL® model.

**Table 1:** Average inhibition zone radii (MM) using the agar diffusion bioassay for various nisin concentrations

Nisin concentration (μg mL^−1^)	Inhibition zone radius (mm)
0.625	3.44 ± 0.08
0.312	2.60 ± 0.12
0.156	1.85 ± 0.19
0.078	1
0.039	1
0.019	1
0.009	1

1 No zone of inhibition was detected.

### Determination of critical time (*T*_c_) for growth of *Micrococcus luteus*

**Figure 3:**
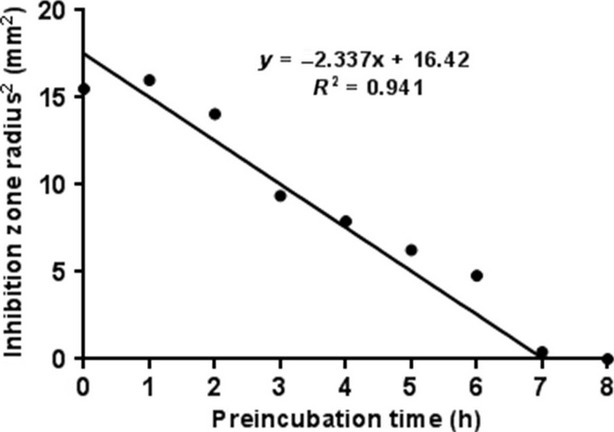
Effect of initial incubation time of nisin at 30°C on the square of inhibition zone radius.

Linton (1958) found that when the agar seeded with *Klebsiella pneumonia* was incubated for a given time ‘*t*’ before adding the antibiotic (1000 μg streptomycin), the size of the inhibition zones reduced. This decrease in inhibition zone size continued until *t* = *T*_c_, at which time no inhibition zones were detected. The square of the inhibition zone radii (*z*^2^) was plotted against initial incubation time (*t*) as shown in Fig.3. A linear relationship was found between the square of the inhibition zone radii (*z*^2^) and incubation time (*t*). The *T*_c_ for *M. luteus* was taken as time corresponding to a *z* value of zero (corresponding to no inhibition zone). In our study, *T*_c_ was experimentally determined to be 7.03 h.

In order to verify the above result, a confirmatory test was conducted wherein the color of the agar plates as described by the *b* Hunter color value (degree of yellowness) was measured every hour when the agar plates were incubated at 30°C, until a significantly higher ‘*b* value was obtained compared to the blank plate (0 h incubation). The average and standard deviation of measurements from five plates are shown in Table2. The Hunter *b* value at time 0 was 1.897, and remained approximately the same up to 6 h of incubation. After 7 h of incubation, the plate was found to have significantly higher (*P* = 0.002) degree of yellowness compared to the plate at 0 h incubation time when a two sample *t*-test was used (*α* < 0.05). This indicates significant growth of *M. luteus* leading to the production of yellow pigmented colonies. Thus, the critical time of 7 h obtained experimentally would indeed have significant bacterial growth in the plate to allow for the appearance of an inhibition zone.

**Table 2:** Hunter color value *b* (degree of yellowness) in seeded agar plates incubated for various times AT 30°C

Incubation time (h)	*b* value^1^
0	1.897 ± 0.17^a^
1	1.864 ± 0.31^a^
2	1.871 ± 0.40^a^
3	1.897 ± 0.33^a^
4	1.956 ± 0.18^a^
5	1.971 ± 0.21^a^
6	2.127 ± 0.20^a^
7	3.011 ± 0.24^b^

1 Mean of five plates.

Means with different superscripts within a column indicate significant difference (*P* < 0.05).

**Figure 4:**
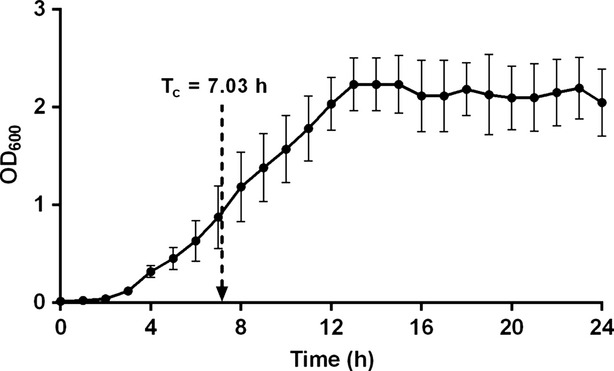
Growth curve for *Micrococcus luteus* in nutrient broth at 30°C.

It has been reported that the critical time of the test organism typically appears during the log phase of the microbial growth curve (Cooper and Linton 1952; Cooper et al. 1958). In order to verify that *M. luteus* is in the log phase at *T*_c_, a growth curve was experimentally generated which is shown in Fig.4. The experimentally determined critical time of 7.03 h, corresponds to the log phase of growth. At this time, the optical density (OD) of the broth measured at 600 nm was 0.6. Based on our calibration curve for *M. luteus* (data not shown), this corresponds to a bacterial population of 2.79 × 10^8^ CFU mL^−1^.

Several researchers (Cooper and Linton 1952; Cooper et al. 1958) have shown that the critical time of the test organism can be approximately related to its lag period (*L*) and generation time (*G*) when grown under standard conditions. Their model was verified experimentally with indicator organisms *Staphylococcus aureus* (Cooper et al. 1958) and *Klebsiella pneumonia* (Linton 1958) against the antibiotic streptomycin. They proposed that the critical time (*T*_c_) for visual appearance of bacterial growth could be described by eq. 5.

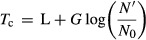
5

where,

*L* = lag period (h)

*G* = generation time (h)

*N*_0_ = initial number of microorganisms in the inoculum (CFU mL^−1^)

*N*′ = number of microorganisms at the *T*_c_ (CFU mL^−1^)

From the growth curve (Fig.4), the lag period (*L*) was determined to be 2 h. Generation time (*G*) can be calculated from this growth curve as the time required for the optical density (OD) of the broth to double. From the growth curve data of OD of 0.8 at 6.8 h, and OD of 0.4 at 4.5 h, the generation time (*G*) of *M. luteus* was calculated to be 2.3 h. The *N*_0_ and *N*′ values were determined from the growth curve to be 10^7^ CFU mL^−1^ and 2.79 × 10^8^ CFU mL^−1^, respectively. Then using eq. 5, the value of *T*_c_ was calculated to be 5.32 h, whereas our experimentally determined *T*_c_ was 7.03 h.

This discrepancy between the experimentally determined *T*_c_ and that predicted by eq. 5 can be attributed to variations in media compositions used in our agar (Cooper et al. 1958). Moreover, the diffusion profiles of nisin obtained from the COMSOL® model showed minimal differences at *T*_c_ = 5.32 h versus *T*_c_ = 7.03 h. For example, at the highest tested initial nisin concentration of 125 μg mL^−1^, the model estimated inhibition zone radius was 10.7 mm at *T*_c_ = 7.03 h as compared to 10.5 mm at *T*_c_ = 5.32 h. Hence the experimentally determined value of 7.03 h was used for subsequent determinations of inhibition zone radii from the computational model.

### Mathematical modeling

**Figure 5:**
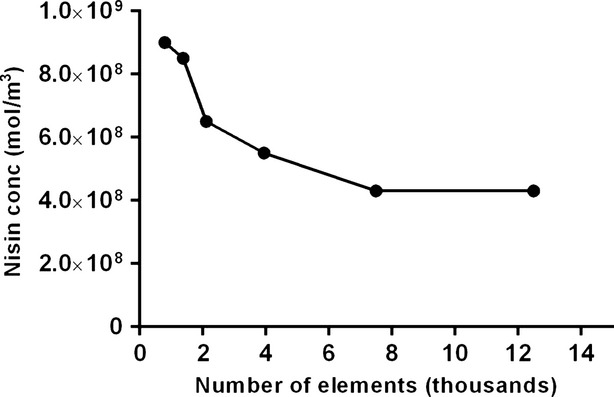
Effect of number of elements on the predicted final nisin concentration (at 10 mm radial distance, for initial nisin concentration of 125 μg mL^−1^).

The element size required for developing the computational model was first determined by generating a preliminary model for an initial nisin concentration of 125 μg mL^−1^. The mesh was continually refined and the nisin concentration profile at the end of the diffusion process (48 h at 4°C followed by 48 h at 30°C) was compared for each simulation run. Figure5 shows the effect of number of elements on the nisin concentration at 10-mm radial distance for ease of comparison. The minimum number of tetrahedral elements that produced convergent results was determined as 7495, and was used for subsequent simulations. The mesh statistics are shown in Table3.

**Table 3:** Mesh description for the computational model

Element type	Number of elements
Tetrahedral	7495
Triangular	3134
Edge	364
Vortex	22
Maximum element size (mm)	0.01
Minimum element size (mm)	0.0018

**Figure 6:**
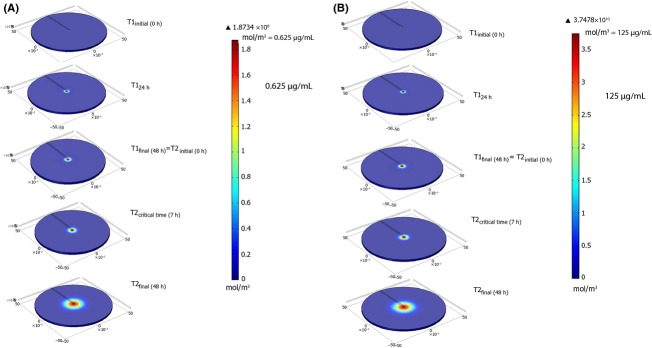
Surface plots of predicted nisin concentration in the agar plate for initial nisin concentrations of (A) 0.625 μg mL^−1^ and (B) 125 μg mL^−1^ at different times. T1: 4°C incubation temperature, T2: 30°C incubation temperature.

Figure6A shows the model-predicted concentration profiles of nisin within the agar plate at different times for an initial nisin concentration of 0.625 μg mL^−1^ (1.8734 × 10^8^ mol m^−3^). T1 indicates the first temperature (4°C) which is the initial diffusion period of the assay, and T2 represents the second temperature (30°C) at which the agar plates are incubated. The figure shows the simulated radial diffusion of nisin as the agar plate is incubated at T1 for 48 h, followed by incubation at T2 for another 48 h. The color in the figure represents the concentration of nisin within the agar in units of mol m^−3^. A color bar legend is shown next to the figure. Blue represents zero nisin concentration. Green to yellow represents intermediate nisin concentrations. Red represents the highest nisin concentration in the agar, which would be equal to the initial nisin concentration in the well. At time point zero, initial nisin concentration in the well is 0.625 μg mL^−1^, and the nisin concentration elsewhere is zero. As the nisin diffuses, the concentration of nisin in the well decreases while the concentration in the area surrounding the well increases radially, as shown in Fig.6A at 24 and 48 h. After 48 h, the diffusivity (D) of nisin changes in value from D_4°C_ to D_30°C_. The diffusivity of nisin is higher at the higher temperature and is known to follow an Arrhenius type relation (Sebti et al. 2004). Thus, the diffusion of nisin from the well is faster at T2 as can be seen from the nisin concentration profile at the critical time (7 h) and at the end of incubation (48 h).

Figure6B shows a similar concentration profile of nisin within the agar plate at different times for an initial nisin concentration of 125 μg mL^−1^ (3.7478 × 10^10^ mol m^−3^). Similar diffusion profiles were constructed for all other initial nisin concentrations (1.25, 2.50, 6.25, 12.50, 18.75, 25, 50, and 75 μg mL^−1^).

**Figure 7:**
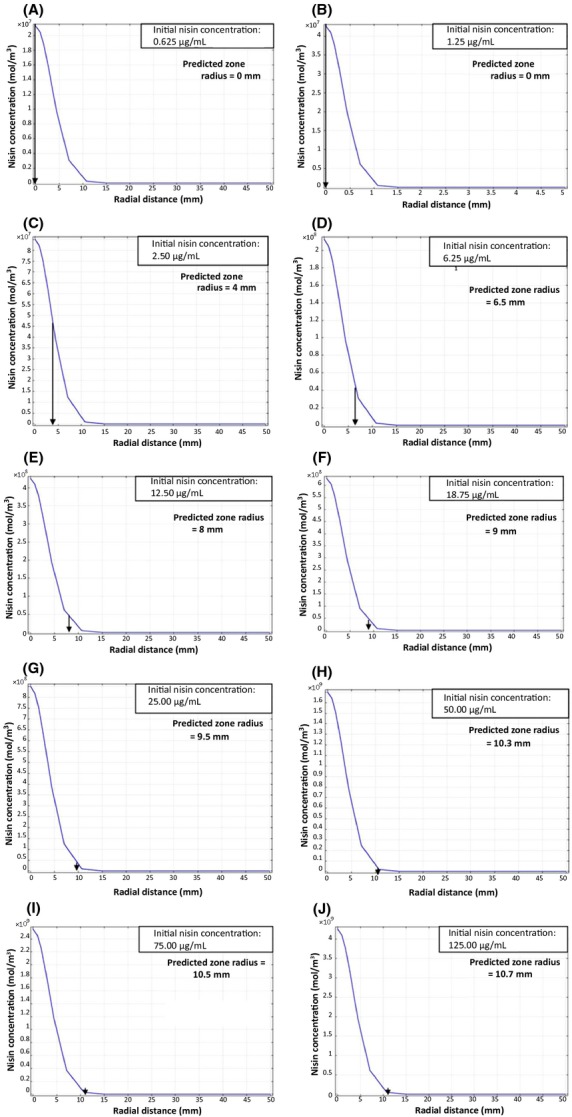
Effect of radial distance on predicted nisin concentration at critical time (*T*_c_) for initial nisin concentrations of (A) 0.625 μg mL^−1^ (B) 1.25 μg mL^−1^ (C) 2.50 μg mL^−1^ (D) 6.25 μg mL^−1^ (E) 12.50 μg mL^−1^ (F) 18.75 μg mL^−1^ (G) 25 μg mL^−1^ (H) 50 μg mL^−1^ (I) 75 μg mL^−1^ (J) 125 μg mL^−1^. The arrow on *x*-axis indicates model-predicted inhibition zone radii where the predicted concentration of nisin = MIC of nisin (4.651 × 10^7^ mol m^−3^).

In order to determine the zone of inhibition, the diffusion profile of nisin across the plate at the critical time (7 h) of incubation at 30°C was used. A 2D plot of nisin concentration versus location on the plate was plotted at the critical time (7 h) for all initial nisin concentrations (Fig.7). From this plot, the distance from the center of the well to the point where the nisin concentration was equal to the MIC, was determined to be the inhibition zone radii. This was determined for each initial nisin concentration.

**Figure 8:**
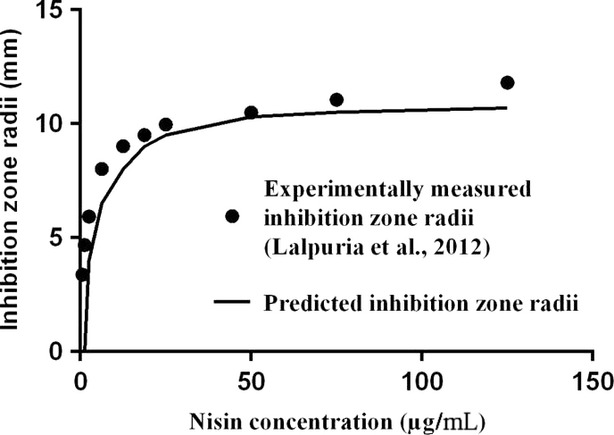
Comparison between experimentally measured (Lalpuria et al. 2012) and model-predicted inhibition zone radii.

The predicted inhibition zones obtained from the FEM model were compared to the experimentally measured zones reported by Lalpuria et al. (2012) (Fig.8). Good agreement was found between experimental and predicted values in the tested range of nisin concentrations (0.625–125 μg mL^−1^) as evidenced by an *R*^2^ value of 0.984. This indicates that the current system can be explained by Fickian diffusion.

**Figure 9:**
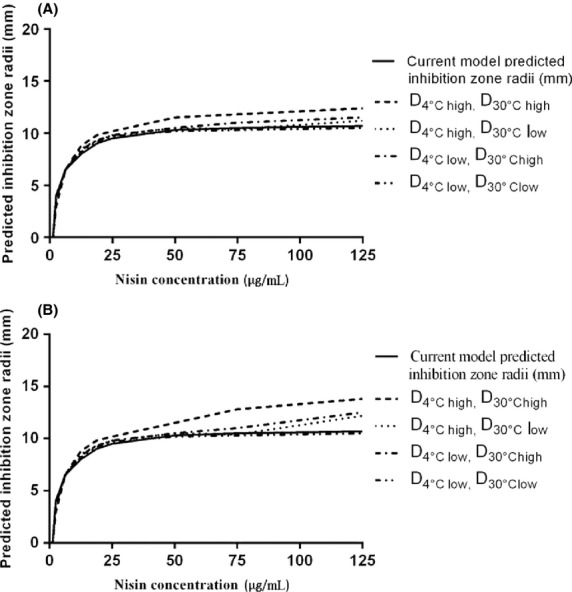
Sensitivity analysis of diffusivity values on predicted inhibition zone radii with (A) 20% increase in diffusivity values (D_4°C high_ = 2.38 × 10^−11^, D_30°C high_ = 1.89 × 10^−10^) or 20% decrease in diffusivity values (D_4°C low_ = 1.58 × 10^−11^, D_30°C low_ = 1.26 × 10^−10^). (B) One order-of-magnitude increase in diffusivity values (D_4°C high_ = 1.98 × 10^−10^, D_30°C high_ = 1.58 × 10^−9^) or one order-of-magnitude decrease in diffusivity values (D_4°C low_ = 1.98 × 10^−12^, D_30°C low_ = 1.98 × 10^−11^).

In developing our diffusion models, the diffusivity values for nisin were assumed to be similar to those reported in the literature for 3% agarose gels (Sebti et al. 2004). Our system consisted of 1% agar gels, based on the method developed by Lalpuria et al. (2013). Sebti et al. (2004) showed that increasing the agarose content from 3% to 6% did not have any effect on nisin diffusion. A sensitivity analysis was carried out to assess the effect of changing nisin diffusivity values on predicted inhibition zone radii values. Figure9a shows the effect of a 20% decrease or increase in nisin diffusivity values (D_4°C_ and D_30°C)_ on the predicted inhibition zone radius. At an initial nisin concentration of 125 μg mL^−1^, when both the diffusivity values (D_4°C_ and D_30°C)_ were changed by +20%, the predicted inhibition zone radii were 15.8 % higher. Figure9b shows the effect of one order-of-magnitude decrease or increase in nisin diffusivity values (D_4°C_ and D_30°C)_ on the predicted inhibition zone radius. At an initial nisin concentration of 125 μg mL^−1^, the predicted inhibition zone radii increased by up to 28.9% when both the diffusivity values (D_4°C_ and D_30°C)_ were increased by one order of magnitude. Changing the diffusivity of nisin at 30°C resulted in larger changes in predicted inhibition zone radii compared to changing the diffusivity at 4°C. This is due to lower diffusivity value of nisin at 4°C. Overall, the sensitivity analysis data show that the predicted inhibition zone radii does not change appreciably even with a one order-of-magnitude change in diffusivity values.

## Conclusions

A diffusion-based finite element model was developed to predict the radius of the inhibition zone in a two-temperature agar diffusion assay using the experimentally determined MIC of nisin against *M. luteus* and the critical time for growth of *M. luteus*. Good agreement (*R*^2^ = 0.984) was obtained between model-predicted and experimentally determined values as reported by Lalpuria et al. (2012). This model can be used as an alternative method to develop standard curves for antimicrobial assays, resulting in savings in time when determining unknown concentrations of antimicrobials. This approach can also be extended to quantify other antimicrobials.

## Conflict of Interest

None declared.
